# The Antiviral Activity of Approved and Novel Drugs against HIV-1 Mutations Evaluated under the Consideration of Dose-Response Curve Slope

**DOI:** 10.1371/journal.pone.0149467

**Published:** 2016-03-01

**Authors:** Shuai Chang, Daomin Zhuang, Wei Guo, Lin Li, Wenfu Zhang, Siyang Liu, Hanping Li, Yongjian Liu, Zuoyi Bao, Jingwan Han, Hongbin Song, Jingyun Li

**Affiliations:** 1 Institute of Disease Control and Prevention, Academy of Military Medical Science, Beijing, China; 2 State Key Laboratory of Pathogen and Biosecurity, Institute of Microbiology and Epidemiology, Academy of Military Medical Science, Beijing, China; 3 NO. 201 hospital, Liaoyang, Liaoning, China; Centro de Biología Molecular Severo Ochoa (CSIC-UAM), SPAIN

## Abstract

**Objectives:**

This study was designed to identify common HIV-1 mutation complexes affecting the slope of inhibition curve, and to propose a new parameter incorporating both the IC50 and the slope to evaluate phenotypic resistance.

**Methods:**

Utilizing site-directed mutagenesis, we constructed 22 HIV-1 common mutation complexes. IC50 and slope of 10 representative approved drugs and a novel agent against these mutations were measured to determine the resistance phenotypes. The values of new parameter incorporating both the IC50 and the slope of the inhibition curve were calculated, and the correlations between parameters were assessed.

**Results:**

Depending on the class of drug, there were intrinsic differences in how the resistance mutations affected the drug parameters. All of the mutations resulted in large increases in the IC50s of nucleoside reverse transcriptase inhibitors. The effects of the mutations on the slope were the most apparent when examining their effects on the inhibition of non-nucleoside reverse transcriptase inhibitors and protease inhibitors. For example, some mutations, such as V82A, had no effect on IC50, but reduced the slope. We proposed a new concept, termed IIP_atoxic_, on the basis of IC50, slope and the maximum limiting concentrations of the drug. The IIP_atoxic_ values of 10 approved drugs and 1 novel agent were calculated, and were closely related to the IIP_max_ values (r > 0.95, *p* < 0.001).

**Conclusions:**

This study confirms that resistance mutations cannot be accurately assessed by IC50 alone, because it tends to underestimate the degree of resistance. The slope parameter is of very importance in the measurement of drug resistance and the effect can be applied to more complex patterns of resistance. This is the most apparent when testing the effects of the mutations on protease inhibitors activity. We also propose a new index, IIP_atoxic_, which incorporates both the IC50 and the slope. This new index could complement current IIP indices, thereby enabling predict the efficacy of pre-clinical drugs for which human pharmacokinetic is not available.

## Introduction

Over the last two decades, advances in antiretroviral therapy have revolutionized the management of human immunodeficiency virus (HIV) and the control of HIV epidemics [1, 2]. Despite these advances, however, some factors, such as drug resistance and rebound viremia under suboptimal treatment conditions [3–8] may lead to treatment failure. Thus, precise quantification of the activity of antiretroviral drugs and drug regimens against drug resistant variants is essential in choosing maximally active drugs and developing newer drugs with high activity and functionality.

Several pharmacodynamic properties are used to determine the activity of a drug. The currently standard measure is the IC50, the concentration of drug required for 50% inhibition *in vitro*. Furthermore, resistance and resistant mutants are typically identified by fold change in IC50 relative to that of the wild-type (WT) strain [9, 10]. The IC50, however, represents only a single point on the dose-response curve and tends to be a poor reflection of antiviral activity among drug classes at higher drug concentrations. For example, recent studies reported that pharmacodynamic parameters such as the IC50 do not distinguish protease inhibitors (PIs) and non-nucleoside reverse transcriptase inhibitors (NNRTIs) from relatively less active nucleoside reverse transcriptase inhibitors (NRTIs) [11–15]. Clinically, antiretroviral drugs are used at concentrations substantially above the IC50, and clinical outcomes may generally depend on whether 99% inhibition is achieved. Indeed, predicting concentrations of antiretroviral drugs based solely on the IC50 when the shape of the dose-response curve is known may result in the loss of important pharmacologic principles underlying the curve. If a drug has a steep dose-response curve, defined as a high slope, a small change in drug concentration can have a significant effect [14], which may push inhibition to less than 100% and lead to an unfavorable outcome. Thus, the slope of the dose-response curve is an important, but generally neglected parameter of antiviral activity [11–17].

Furthermore, it is implicit in current studies that resistance mutations shift dose-response curves to the right alone without affecting their slope [4, 9, 10]. It is noteworthy that resistance mutations that reduce the slope of the dose-response curve may result in substantial drug resistance. To date, however, the effects of resistance mutations on the slope have not been brought into the laboratory monitoring as a standard parameter when evaluating antiviral activity of a drug. Currently, the wide coverage of highly active antiretroviral therapy (HAART) has led to HIV-1 mutation complexes becoming the most frequent patterns in treating HIV-infected individuals. It is unclear that the effect of resistance mutation complexes on antiretroviral activity under the consideration of dose-response curve slope.

Instantaneous inhibitory potential (IIP), which is dependent on IC50, slope, and *in vivo* drug concentration, is a more accurate pharmacodynamic measure of antiviral activity than current parameters such as IC50 alone [11]. However, it is impossible to determine the plasma concentrations of novel, pre-clinical drugs. There is a need to add a parameter to the IIP indices to allow the assessment of drugs in the absence of *in vivo* plasma concentration.

This study was therefore designed to evaluate the effects of common HIV-1 resistance mutation complexes on the IC50 and slope, and to propose a parameter that incorporates both IC50 and slope to determine the efficacy of pre-clinical antiretroviral drug candidates.

## Materials and Methods

### Antiretroviral compounds

The antiretroviral drugs used in this study included zidovudine (ZDV, AZT), lamivudine (3TC), indinavir (IDV), nelfinavir (NFV), saquinavir (SQV), and ritonavir (RTV), all from Sigma-Aldrich Co. (St. Louis, MO, USA); and didanosine (ddI), stavudine (d4T), nevirapine (NVP), and efavirenz (EFV), all from Shanghai Desano Chemical Pharmaceutical Development Co., Ltd. (Shanghai, China). All of these drugs are used commonly in China for antiretroviral treatment. DG35 was a new PI, provided by Hesi Scientific and Technology Ltd (Xi’an, Shaanxi, China).

All of these drugs were dissolved in dimethyl sulfoxide (DMSO) and stored at -20°C. Drugs were serially diluted, such that the final concentration of DMSO in cell culture medium was 0.5%.

### Cells and viruses

The human T-cell line MT-2 [18] was maintained in RPMI 1640 medium (Gibco, Gaithersburg, MD, USA). Human embryonic kidney 293T cells [19] are adenovirus-transformed human embryonic kidney cells, and TZM-bl cells (JB53BL-13) [20] contain luciferase and β-galactosidase genes under the control of HIV Tat expression. Both cell lines were cultured in Dulbecco’s Modified Eagle’s Medium (DMEM) (Gibco, Gaithersburg, MD, USA). All of these cell lines were kindly provided by Dr. Lu of the Laboratory of Nucleic Acid Vaccines at the University of Massachusetts Medical School (Worcester, MA, USA) in 2005. Culture media were supplemented with 10% fetal bovine serum (FBS) (Gibco, Gaithersburg, MD, USA), and cells were cultured at 37°C and 5% CO_2_. HIV-1_NL4-3_ was initially obtained from Dr. Lu in 2006 and long-term stored in our laboratory.

### Construction of HIV-1 mutants

Variant viruses bearing single or multiple amino-acid substitutions, which are commonly found in Chinese HIV drug resistance surveillance programs, were obtained by site-directed mutagenesis on a pNL4-3 wild-type background. In brief, because of the large size of the pNL4-3 plasmid (~15 Kb), the entire protease (PR) (codons 1–99) and reverse transcriptase (RT) (codons 1–312) regions of this plasmid were amplified and ligated into the pMD18T vector (TaKaRa Biotechnology Co., Ltd., Dalian, Liaoning, China), followed by site-directed mutagenesis using the Phusion^™^ Site-directed Mutagenesis Kit (New England Biolabs, Ltd., Beijing, China). M46I, I54V, V82A, M46I\N88S, G48V\I54V, M46I\V82T\I84V, and G48V\I54V\V82A mutations were introduced into the PR coding region, and K103N, Y181C, K103N\Y181C, K101Q, K101Q\Y181C, K101Q\H221Y, K101Q\H221Y\Y181C, V179E\T215Y, V179E\Y181C\T215Y, V179E\H221Y\T215Y, V179E\Y181C\H221Y\T215Y, K103N\Y181C\T215Y, K103N\H221Y\T215Y, K103N\H221Y\Y181C\T215Y, and M41L\L210W\T215Y\K103N\K238T mutations were introduced into the RT coding region. The ligations harboring the desired mutations were digested with the restriction enzymes *SphI* and *AgeI* (New England BioLabs, Ltd., Beijing, China), and the digested targeted segments were cloned into the pNL4.3-Δ(*SphI*-*AgeI*) vector. These plasmids were subsequently transfected into HEK293T cells using Lipofectamine^®^ 2000 (Life Technologies Co., Carlsbad, CA, USA), according to the manufacturer’s instructions. Culture supernatants were harvested 2–3 days after transfection, stored at -80°C, and sequenced to confirm the presence of the desired mutations.

### *In vitro* drug susceptibility assay

For experiments testing RT inhibitors, triple serial dilutions spanning empirically determined ranges for each drug were added to wells of 384-well plates. TZM-bl cells (10,000 cells/well) were infected with recombinant virus at an MOI of 0.02 in plates containing pre-plated antiretroviral drugs. After 48 h, the expression of the luciferase reporter gene was measured using a Bright-Glo Luciferase Assay (Promega Co., Madison, WI, USA).

As the inhibitory effect of PIs cannot be detected in TZM-bl cells after 48 h, the protocol for PI susceptibility assays was based on a modification of the reporter gene assay for determining antiretroviral activity. During the first round of infection, 200 TCID50s of each viral stock were used to infect 13,500 MT-2 cells (MOI, 0.01) in diaphanous 384-well plates containing 3-fold serial dilutions of each tested PI. After 72 h in culture, 20 μL of supernatant containing *de novo* produced viral particles was transferred to new TZM-bl cultures (12,000 cells/well) in black 384-well plates. HIV-1 infection in this second round was monitored by measuring β-galactosidase expression in infected target cells 24 h after infection.

### *In vitro* cellular toxicity assay

The potential cellular toxicities of drugs in MT-2 cells were determined by measuring cellular ATP levels in the presence of various concentrations of these compounds. MT-2 cells (13,500 cells/well) were cultured with each compound at 37°C and 5% CO_2_. After 72 h, CellTiter-Glo reagent (Promega Co., Madison, WI, USA) was added to each well, and chemiluminescence was measured. The maximum nontoxic concentration was defined as the concentration of inhibitor that had no effect on cellular ATP levels.

### Analysis of dose-response curves

Percent inhibition was calculated as [1 − (virus production in the presence of drug / virus production in the absence of drug)] × 100. The IC50 and slope of the inhibition curve of each inhibitor were determined by fitting the inhibition curves to the data using nonlinear regression analysis to generate a four parameter sigmoid dose-response equation (GraphPad Prism, version 6.02). This step was performed in triplicate for duplicate plates of each concentration of antiretroviral drug. The mean IC50 and slope were calculated using all of the replicates for each virus and were expressed as mean ± standard deviation. The slope parameter is analogous to the Hill coefficient, which determines the degree of cooperativeness of the ligand binding to the enzyme or receptor [21]. A coefficient of 1 indicates that the affinity of the enzyme for a ligand is independent on whether or not other ligands are already present on the same enzyme. When the coefficient applies to the binding of anti-HIV drugs to the relevant HIV-1 enzymes, a coefficient of 1 indicates that there is only a single drug target mediating a step in the life cycle [22].

IIP, which incorporates IC50, slope, and drug concentration, was used to better evaluate the antiviral activity of drugs [11]. IIP was calculated using the equation: *IIP = log [1 + (D / IC50)* ^*m*^*]* (1), where D is the drug concentration and m is the slope of the dose-response curve. The results were expressed as fold changes in IC50 and fractional changes in slope and IIP. The fold change in IC50 reflected the IC50 of a particular drug to a mutant virus relative to the IC50 of the same drug to a wild-type reference virus (HIV_NL4-3_). The fractional change in slope was computed as: *1 − (m*_*mutant*_ */ m*_*NL4-3*_*)* (2); and the fractional change in IIP was computed as: *1 − (IIP*_*mutant*_ */ IIP*_*NL4-3*_*)* (3).

### Statistical analyses

The mean and standard deviations for IC50, slope, and IIP were calculated using Microsoft Office Excel 2013 software. Pearson’s correlation coefficient was used to determine correlations between parameters. All statistical analyses were performed using SPSS version 16.0 software, and *p*-values less than 0.05 were considered statistically significant.

## Results

### Analysis of dose-response curves

**Fig 1:**
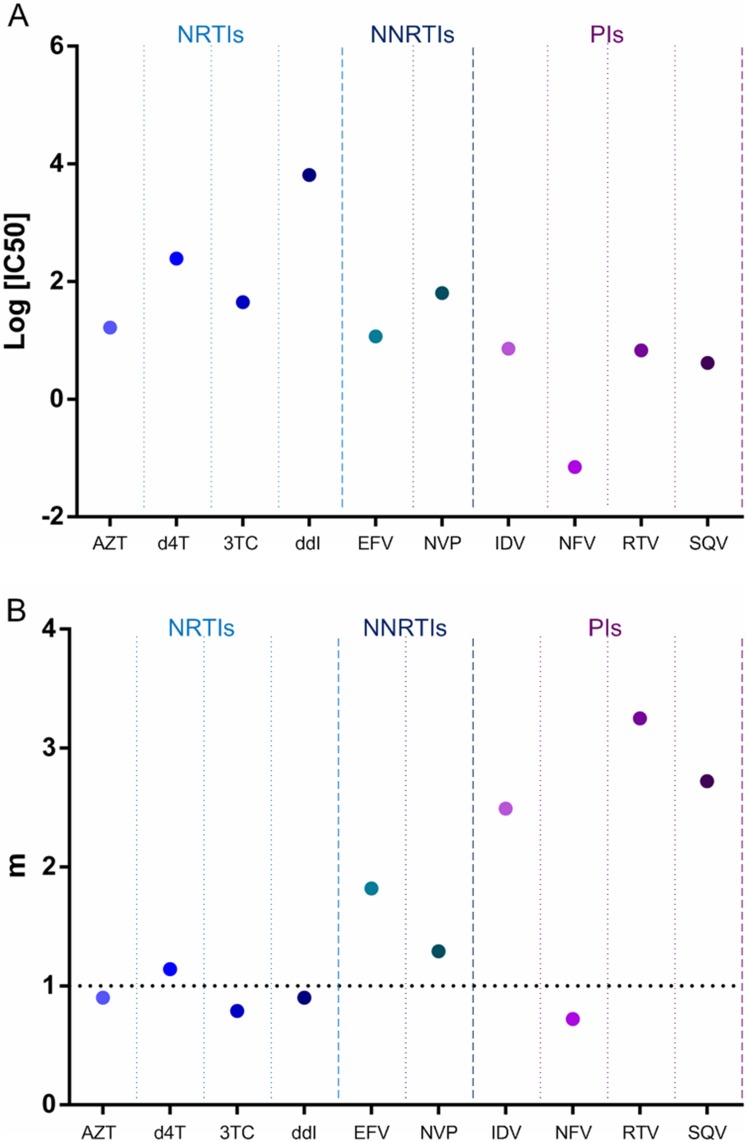
(A) IC50s and (B) slopes of three classes of antiretroviral drugs against HIV_NL4-3_, as determined during multiple-round infectivity assays.

Drug susceptibility was assayed based on the MT-2/TZM-bl cells assay system. Fig 1 shows the IC50 and slope of various approved drugs for wild-type HIV_NL4-3_. Depending on drug class, there were intrinsic differences in the dose-response curves, both in their location (IC50) and shape (slope). The degree of IC50 varied widely, and the slope values for NNRTIs and PIs, except for NFV, were above 1, although those for most NRTIs were less than 1.

### Effect of RT inhibitor associated mutations on IC50 and slope

**Fig 2:**
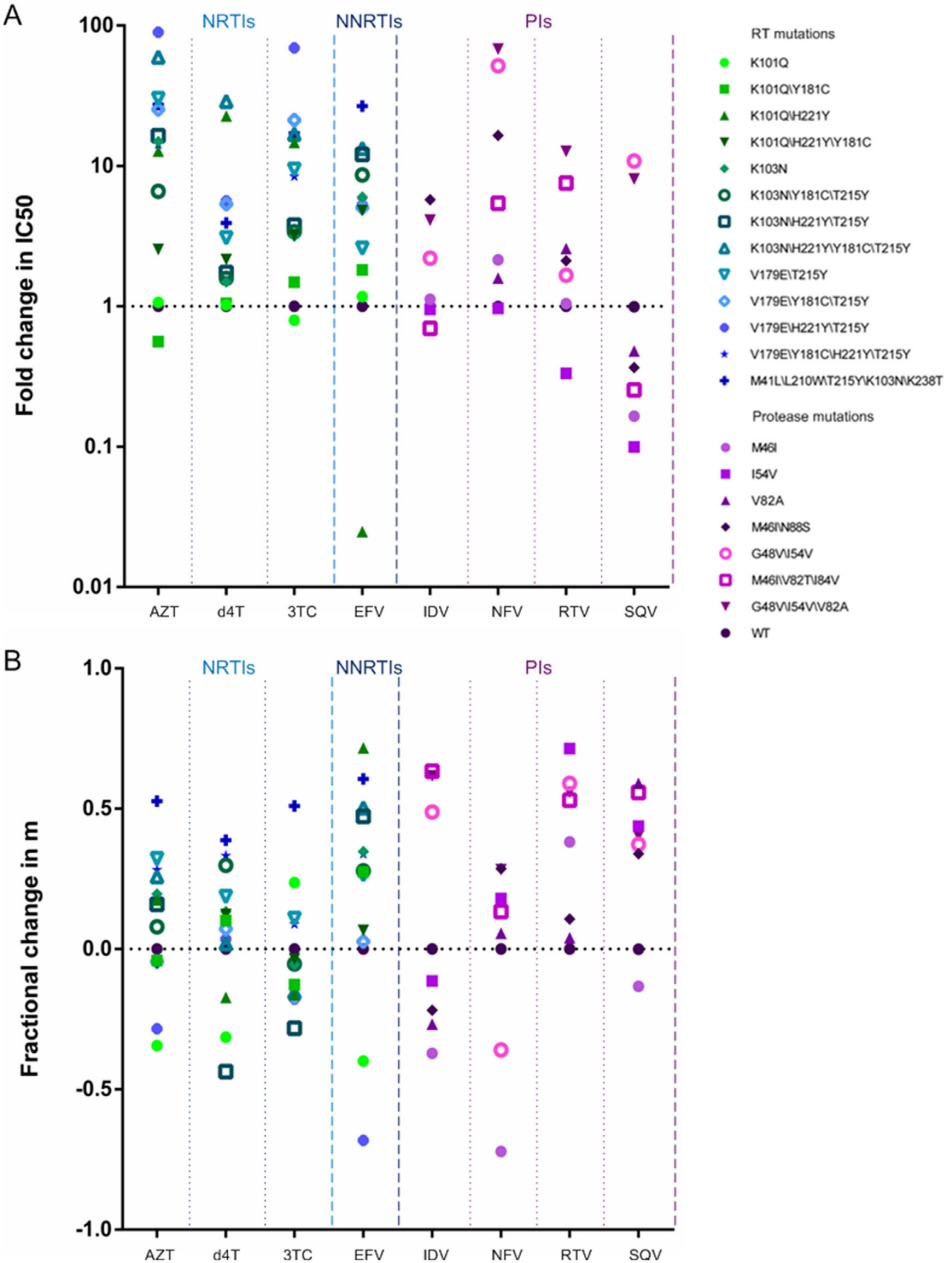
Effects of various resistance mutations on (A) IC50 and (B) slope, as calculated from dose-response curves. Fold changes in IC50 for mutants were relative to those for the wild-type virus, and fractional changes in slope were computed using equation (2). The drugs tested are grouped by class: NRTIs, NNRTI, and PIs. Within each class, different shapes indicate the various mutants.

**Fig 3:**
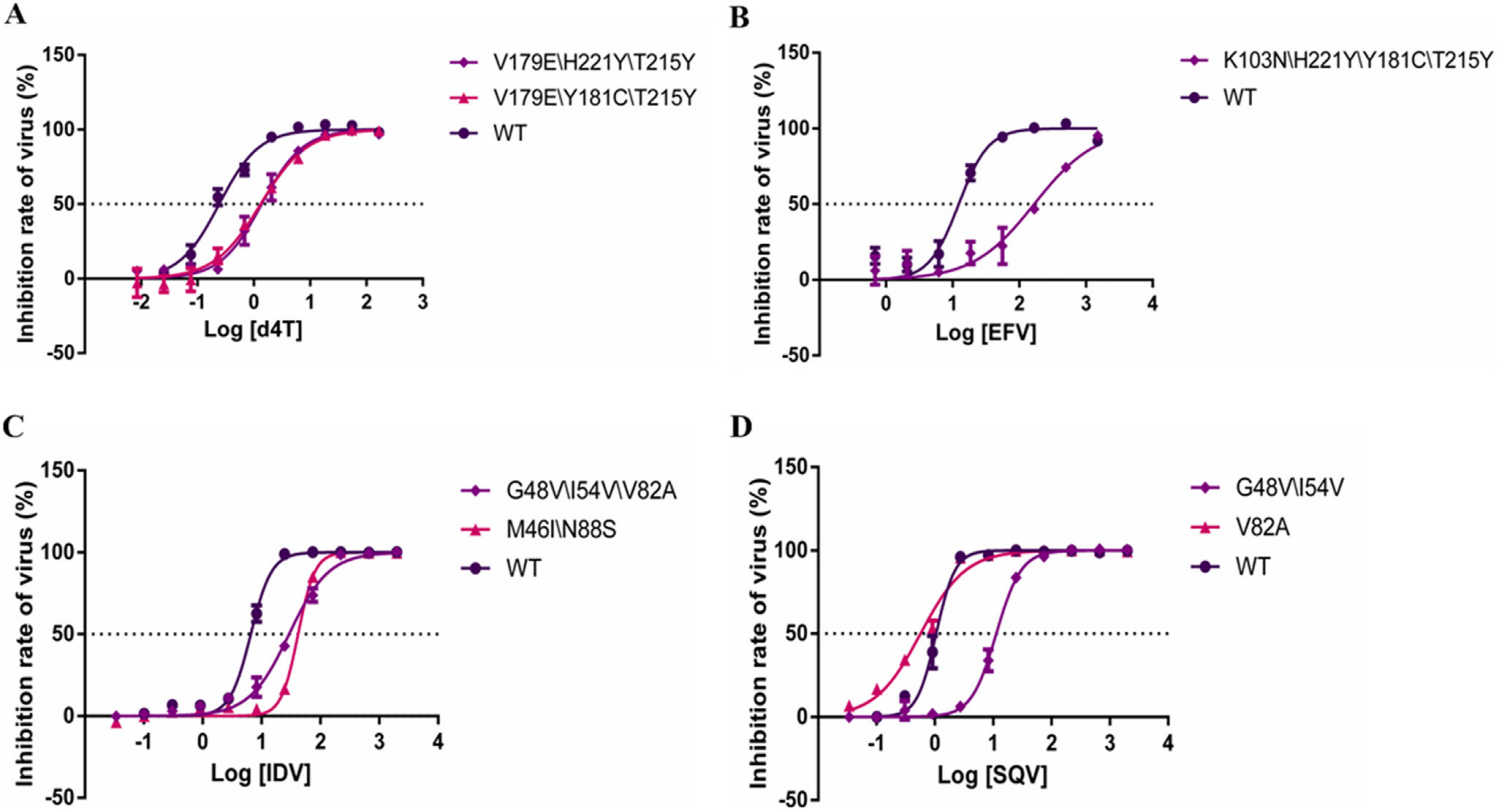
Curves of the dose-response of selected drugs. x axes indicate log of drug concentration (nM), y axes indicate the inhibitor rate of virus (%). (A) is the curve of the dose-response of 3 viruses (V179E\H221Y\T215Y, V179E\Y181C\T215Y and WT _NL4.3_) in d4T. (B) is the curve of the dose-response of 2 viruses (K103N\H221Y\Y181C\T215Y and WT _NL4.3_) in EFV. (C) is the curve of the dose-response of 3 viruses (G48V\I54V\V82A, M46I\N88S and WT _NL4.3_) in IDV. (D) is the curve of the dose-response of 3 viruses (G48V\I54V, V82A and WT _NL4.3_) in SQV.

Utilizing site-directed mutagenesis, we constructed viruses bearing common mutations that confer drug resistance. Fig 2A and 2B show fold changes in IC50 and fractional changes in slope, respectively, for mutants relative to the wild-type virus. All of these mutations resulted in large increases in the IC50s for NRTIs, without affecting the slope of the curves (Fig 3A). Furthermore, similar results were also observed in the curves of some NNRTIs. For example, the inhibition curve of the K103N\H221Y\T215Y mutation was shifted toward a higher drug concentration for EFV (12.09-fold), with no alterations in shape. However, the K103N\H221Y\Y181C\T215Y mutation led to an intermediate shift in IC50 but a marked reduction in slope (Fig 3B). Although the virus harboring the K101Q\H221Y mutation was found to be hyper-susceptible to EFV, the slope of the curve was significantly decreased. This effect of resistance would not be found in assays based solely on fold changes in IC50.

### Effect of PI associated mutations on IC50 and slope

The effect of resistance mutations on IC50 and slope was more evident for PIs than for NRTIs and NNRTIs **(**Table 1, S1 and S2 Tables). As shown in Fig 3C, the triple-mutant G48V\I54V\V82A affected both IC50 and slope for this agent, whereas M46I\N88S reduced the IC50 but not the slope. G48V\I54V\V82A mutations increased the IC50 and reduced the slope for RTV, leading to a marked decrease in RTV susceptibility at concentrations above the IC50. The V82A mutation, however, had no effect on RTV antiviral activity, and the G48V\I54V mutation reduced the slope but not the IC50. Interestingly, addition of V82A to G48V\I54V increased IC50 68.1-fold, greater than the sum of the effects of each in the presence of NFV. These findings suggested that the interaction between V82A and G48V\I54V was synergistic. Furthermore, Fig 3D showed that the G48V\I54V mutation increased the IC50 without affecting slope of dose-response curve for SQV. While the mutation V82A had no effect on IC50, but reduced the slope of the dose-response curve for this agent.

**Table 1:** Fold changes in IC50 and fractional changes in the slopes of the inhibition curves of various drugs for the selected protease inhibitors resistance mutations.

Mutations	IDV		NFV		RTV		SQV	
	Fold change in IC50 *	Fractional change in slope *	Fold change in IC50 *	Fractional change in slope *	Fold change in IC50 *	Fractional change in slope *	Fold change in IC50 *	Fractional change in slope *
M46I	1.12±0.06	-0.37±0.08	2.15±0.37	-0.72±0.06	1.05±0.32	0.38±0.15	0.16±0.03	-0.13±0.04
I54V	0.95±0.01	-0.11±0.13	0.97±0.01	0.18±0.08	0.33±0.12	0.71±0.11	0.10±0.03	0.44±0.08
V82A	1.09±0.17	-0.27±0.10	1.59±0.33	0.06±0.12	2.58±1.30	0.04±0.01	0.48±0.23	0.59±0.05
M46I\N88S	5.75±0.73	-0.22±0.05	16.54±9.03	0.29±0.02	2.11±0.26	0.11±0.02	0.37±0.04	0.34±0.18
G48V\I54V	2.20±0.71	0.49±0.02	51.78±6.00	-0.36±0.10	1.66±0.05	0.59±0.11	10.87±0.67	0.37±0.01
M46I\V82T\I84V	0.70±0.06	0.63±0.15	5.42±2.11	0.13±0.06	7.56±2.44	0.53±0.15	0.25±0.01	0.56±0.05
G48V\I54V\V82A	4.12±1.90	0.62±0.07	68.14±1.45	0.28±0.14	12.72±1.86	0.56±0.01	8.07±2.54	0.40±0.07
WT ^#^	1.00	0.00	1.00	0.00	1.00	0.00	1.00	0.00

* Values are expressed as means ± standard deviations of three independent experiments.

^#^ WT indicates the wild-type HIV _NL4-3_, as a control.

### Parameters for assessing approved and novel drugs

**Fig 4:**
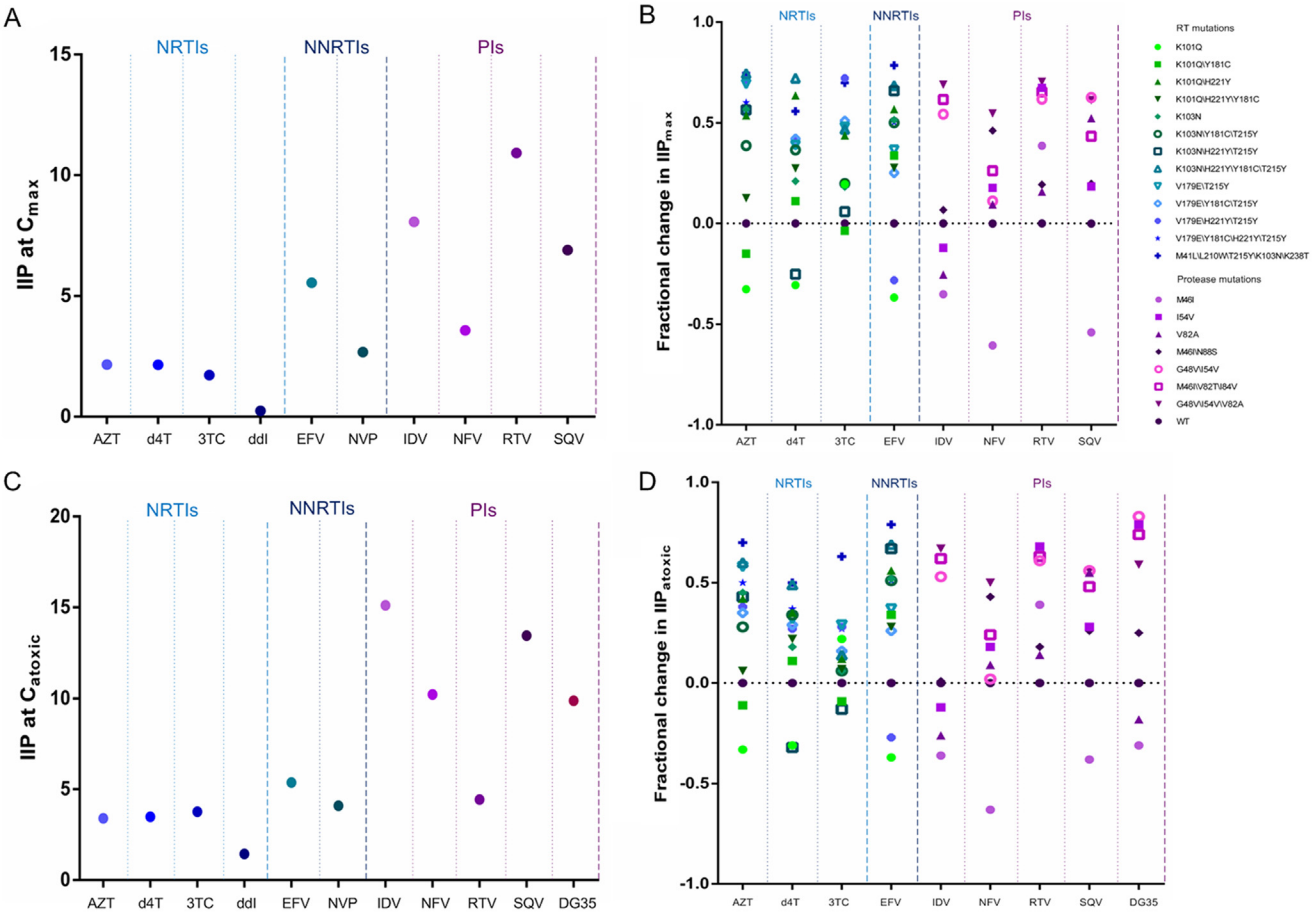
Effect of various viruses on (A, B) IIP_max_ and (C, D) IIP_atoxic_ toward (A, C) HIV_NL4-3_ and (B, D) mutant viruses. IIP was calculated using equation (1), with the concentrations used being peak plasma concentrations for IIP_max_ and the maximum nontoxic concentration for IIP_atoxic_. Fractional changes in IIP were calculated using equation (3). The drugs tested are grouped by class: NRTIs, NNRTI, and PIs. Within each class, different shapes indicate the various mutants.

IIP was calculated using equation (1), which included the IC50, slope and concentration of antiviral drugs. To simulate the real activity *in vivo*, peak plasma concentrations of the drugs were used for the calculations, with the results shown as IIP at C_max_. Fig 4A and 4B show the IIP_max_ values of the approved drugs against various viruses. The values for NNRTIs and PIs were greater than those for NRTIs. Due to the lack of clinical data, we could not calculate the IIP_max_ values for novel drugs. In our pre-clinical evaluation, the maximum nontoxic concentration of each drug was represented as D, and the values were shown as IIP_atoxic_. Both the toxicity and activity of an inhibitor were taken into consideration. Fig 4C and 4D show the IIP_atoxic_ values of the approved drugs and a novel, pre-clinical agent against various viruses. Similar to findings for IIP_max_, the IIP_atoxic_ values of NNRTIs and PIs exceeded those of NRTIs. Specially, the novel PI DG35 exhibited satisfactory activity against both wild-type and drug resistant viruses.

### The relationship between fold changes in IC50 and fractional changes in IIP

A lack of correlation was observed between fold changes in IC50 and fractional changes in IIP_max_ for EFV and PIs (*p* > 0.05 each). Using fractional change in IIP_max_ accounted for the effects of change in slope. Furthermore, fold changes in IC50 did not correlate with fractional changes in IIP_atoxic_ for any of the drugs tested (*p* > 0.05 each), suggesting that IIP_atoxic_ can reflect the effects of change in slope. There were strong correlations (r > 0.95 each) between fractional changes in IIP_max_ and those in IIP_atoxic_ (*p* < 0.001 each) for all drugs tested.

## Discussion

A problem with using IC50 as an indicator of antiviral activity is that it obscures differences in antiviral activity at higher drug concentrations. The slope of the dose-response curve, however, provides a better indication of antiviral activity at high drug concentrations. Slopes can distinguish antiretroviral drugs from different classes with the same IC50 and are intrinsically drug class-specific [11–15]. Thus, slope is an important determinant of antiviral activity. This study showed that the IC50 values varied widely, and that the slopes of most NNRTIs and PIs were higher than those of most NRTIs, in agreement with previous studies [11–15]. This is likely to explain the satisfactory clinical utility of these two classes of antiretroviral drugs, with the most effective combination regimens including an NNRTI or a PI [23]. Currently, the PI monotherapy has been proposed and been undergoing evaluation [24]. The higher slope of PIs may be attributed to the cooperative binding of multiple ligands to a multivalent receptor [22].

Many studies have reported that longer cumulative exposure to HAART correlates with higher rates of HIV resistance in China [25–27]. The wide coverage of HAART has led to complex mutation patterns becoming a serious problem. Currently, HIV-1 mutation complexes have been the most frequent patterns in treating HIV-infected individuals. To illustrate the effect of anti-HIV-1 drug resistance mutations on IC50 and slope, we assessed the activity of 10 widely used drugs against 22 common HIV-1 resistance mutation complexes in China. All of the RT mutations we tested increased the IC50 for NRTIs but had relatively little effect on slope. By contrast, three of the seven PR mutations tested reduced the slope but increased the IC50 of PIs. Specially, our study also showed that there were mutations that affected the slope alone. For example, the findings indicated that the mutation V82A was not a primary mutation for SQV based on the parameter IC50. However, V82A reduced the slope of the dose-response curve, such that SQV activity was markedly reduced at concentrations above the IC50, an effect due to its molecular mechanism [28–30]. Under these conditions, the traditional single-parameter (IC50) may underestimate the degree of resistance, leading to unsatisfactory outcomes in patients.

Taken together, our results revealed that resistance cannot be accurately assessed by IC50 alone, and that the effect of slope can be applied to more complex patterns of resistance. Furthermore, depending on drug class, there were intrinsic differences in the method by which resistance mutations affect drug parameters. The effects of mutants on slope were the most apparent when assessing the effects of mutants on PIs. The differences in the effects of the mutations on the parameters may be a consequence of the different mechanisms of drug inhibition. Mutations may lower enzyme efficiency, which affects the number of PR molecules needed to complete maturation. The exact mechanisms underlying the intrinsic differences in drug resistance are not yet fully understood and need further investigation.

For licensed drugs, the activity mainly depends on the intrinsic pharmacodynamic properties (IC50 and slope) and the pharmacokinetic properties of each drug *in vivo* (the value of D over time) [13]. IIP_max_, a parameter that takes into account the pharmacodynamic and pharmacokinetic properties of an individual drug, may be superior to traditional measures. D values *in vivo*, however, have not yet been determined for some candidate drugs, making their IIP_max_ unknown. The United States Food and Drug Administration (FDA) had recommended conducting systematic resistance studies for promising inhibitors before entering clinical trials. In the present study, we proposed a concept of IIP_atoxic_, with D defined as the maximum nontoxic concentration of a drug based on its chemical structure. Thus, it is reasonable to calculate IIP_atoxic_ on the basis of IC50, slope and the maximum limiting concentrations of promising pre-clinical inhibitors. Like fractional changes in IIP_max_ having no relation with fold changes in IC50, we also observed a poor correlation between fractional changes in IIP_atoxic_ and fold changes in IC50, suggesting that IIP_atoxic_ can account for the effects of changes in slope. Furthermore, strong correlations between fractional changes in IIP_max_ and those in IIP_atoxic_ for all drugs tested were observed (r > 0.95, *p* < 0.001 each). Similar to their IIP_max_ values, the IIP_atoxic_ values of approved NNRTIs and PIs exceeded those of approved NRTIs. Using IIP_atoxic_ values, we found that the novel PI (DG35) showed satisfactory performance against both wild-type and mutant viruses. The ability to calculate IIP_atoxic_ in the absence of plasma concentration suggests its potential use in screening for high-activity inhibitors from a large pool of potential candidates.

## Conclusions

This study confirmed that resistance mutations cannot be accurately assessed by IC50 alone, because it tends to underestimate the degree of resistance. The slope parameter is of very importance in the measurement of drug resistance and the effect can be applied to more complex patterns of resistance. This was the most apparent when testing the effects of mutants on PI activity. We also added a new parameter, IIP_atoxic_, to IIP indices for novel, pre-clinical drugs. The new parameter incorporates both the IC50 and the slope, thereby enabling predict the efficacy of pre-clinical drugs for which human pharmacokinetic is not available.

## Supporting Information

S1 Table Fold changes in IC50 of various drugs for the protease inhibitors resistance mutations. (XLSX)

S2 Table Fractional changes in the slopes of the inhibition curves of various drugs for the protease inhibitors resistance mutations. (XLSX)
